# The capacity of neurological pupil index to predict the absence of somatosensory evoked potentials after cardiac arrest – An observational study

**DOI:** 10.1016/j.resplu.2024.100567

**Published:** 2024-02-03

**Authors:** Meena Thuccani, Sara Joelsson, Linus Lilja, Axel Strålin, Josefin Nilsson, Petra Redfors, Araz Rawshani, Johan Herlitz, Peter Lundgren, Christian Rylander

**Affiliations:** a Department of Molecular and Clinical Medicine, Institute of Medicine, Sahlgrenska Academy, University of Gothenburg, Gothenburg, Sweden; b Anaesthesiology and Intensive Care, Department of Surgical Sciences, Uppsala University, Uppsala, Sweden; c Prehospen – Centre for Prehospital Research, University of Borås, Sweden; d Department of Cardiology, Sahlgrenska University Hospital, Gothenburg, Sweden; e Department of Anaesthesiology and Intensive Care Medicine, Institute of Clinical Sciences, Sahlgrenska Academy, University of Gothenburg, Gothenburg, Sweden; f Department of Anaesthesia and Intensive Care, Karlstad Central Hospital, Karlstad, Sweden; g Department of Clinical Neuroscience, Institute of Neuroscience and Physiology, Sahlgrenska Academy, University of Gothenburg, Gothenburg, Sweden; h Department of Neurology, Sahlgrenska University Hospital, Gothenburg, Sweden; i Department of Clinical Neurophysiology, Institute of Neuroscience and Physiology, Sahlgrenska Academy, University of Gothenburg, Gothenburg, Sweden

**Keywords:** Cardiac arrest, Neurological outcome, Neurological pupil index, Prognostication, Pupillometry, Somatosensory evoked potentials

## Abstract

**Background:**

In neurologic prognostication of comatose survivors from cardiac arrest, two independent predictors of poor outcome are the loss of the Pupillary light reflex (PLR) and the loss of the N20 response from Somatosensory Evoked potentials (SSEP). The PLR can be quantitatively assessed by pupillometry. Both tests depend on the midbrain, in which a dysfunction reflects a severe hypoxic injury. We reasoned that a certain level of defective PLR would be predictive of a bilaterally absent SSEP N20 response.

**Method:**

Neurological Pupil index (NPi) from the pupillometry and the SSEP N20 response were registered >48 h after cardiac arrest in comatose survivors. Clinical data were retrospectively analyzed. A receiver operating characteristic curve was used to evaluate the capacity of NPi to predict bilaterally absent SSEP N20 response. An NPi threshold value resulting in <5% false positive rate (FPR) for bilaterally absent N20 response was identified.

**Results:**

From February 2020 to August 2022, we included 54 patients out of which 49 had conclusive pupillometry and SSEP examinations. The NPi threshold value with FPR < 5% was 3.4, yielding 36% sensitivity (95% CI 18–55) and significantly discriminated between respective groups with preserved and bilaterally absent N20 response to SSEP (*p*-value <0.01).

**Conclusion:**

In this limited cohort, NPi < 3.4 in patients remaining comatose >48 hours after cardiac arrest predicted bilateral loss of the SSEP N20 response with a FPR < 5%. If validated in a larger cohort, an NPi threshold may be clinically applied in settings where SSEP is unavailable.

## Introduction

Severe hypoxic ischemic encephalopathy (HIE), which develops from hours to days after resuscitation, is the primary cause of death among comatose survivors of cardiac arrest.^1^, ^2^, ^3^ Identifying patients with poor neurological outcome among comatose survivors is challenging due to the delayed presentation of the extent of the brain injury. Yet, this assessment of neurologic prognosis is vital to enable adequate decisions to withdraw life-sustaining therapy (WLST).^4^ The internationally recommended model for neurologic examination includes multimodal methods independent of each other.^5^ The aggregated result yields an acceptably low false positive rate (FPR) for the identification of a poor prognosis.^4^, ^6^, ^7^ Two of the tests are robust predictors independently yielding a low FPR when used correctly; the pupillary light reflex (PLR) and Somatosensory Evoked Potentials (SSEP).^8^

Due to standard manual PLR assessment being subjected to several confounding factors, pupillometry has emerged as an aid for reproducible assessment of PLR characteristics associated with poor neurologic outcome.^9^, ^10^, ^11^, ^12^, ^13^ The pupillometer is a handheld automated device with a camera that measures the PLR to a standardized light stimulus. Specifically, a composite measure of the PLR, the Neurological Pupil index (NPi), has been used.^14^ Examination of the cortical SSEP response to median nerve stimulation, registered as the N20 signal, is a robust bedside technique, but it requires special equipment and personnel training.^15^ Presence of the N20 signal indicates thalamocortical connectivity and similar to the loss of PLR after cardiac arrest, bilateral absence of the N20 signal is predictive of poor neurologic outcome with an FPR < 5%.^8^ However, while pupillometry can be readily available at a low cost, only 53% of Swedish intensive care units (ICU) have access to SSEP for neurologic prognostication.^16^

The N20 signal for the cortical SSEP response travels via the medial lemniscus in the midbrain, a neighbouring structure to the Edinger-Westphal nucleus and ocular nerve nucleus, critical for the PLR. Hypothetically, a hypoxic lesion that affects the midbrain may cause an absence of the PLR as well as the N20 response. The midbrain is, however, more resistant to hypoxic injury than the cerebral cortex.^4^ We reasoned that the PLR may be impaired but still preserved at a point of HIE where the cortical SSEP response is lost. If the PLR were examined by pupillometry, NPi values below a certain level would be predictive of bilateral loss of the SSEP N20 response. If so, the technically less demanding pupillometry could be used as a proxy for SSEP in neurologic prognostication after cardiac arrest.

## Material and methods

This study was conducted from February 2020 to August 2022 in a mixed general ICU at Sahlgrenska University Hospital in Gothenburg, Sweden. The hospital is the referral centre for specialized cardiac care for 1.7 million inhabitants in western Sweden. The yearly admittance of resuscitated survivors of cardiac arrest to the general ICU is 50–60 patients. The study was approved by the Swedish Ethical Review Authority (DNR 2019-00823, 2020-00506) and the protocol (Clinical Trials NCT04720482) was published before.^17^ After providing verbal and written information, consent was obtained from patients who regained consciousness or the next of kin when appropriate. Data was handled and stored according to the European General Data Protection Regulation (GDPR). The manuscript was prepared according to the Standards for Reporting of Diagnostic Accuracy Studies (STARD) guidelines.^18^

### Study design

For this non-interventional, observational study, eligible patients were comatose (motor score ≤ 3) survivors of cardiac arrest who underwent routine neurological prognostication, including pupillometry and SSEP, in the ICU. Exclusion criteria were: regained consciousness < 48 hours after cardiac arrest or before examination by SSEP could be performed; pregnancy; intracranial bleeding; traumatic brain injury and palliative care. The patients were included by convenience, depending on availability of the researchers and other logistic factors.

### Clinical protocol

The local standard operating procedure (SOP) for comatose survivors of cardiac arrest included 24 hours of targeted temperature management (TTM) at 36 °C or 37.5 °C using a water mediated surface cooling device (Arctic Sun 2000 TTM; Bard Medical). During these 24 hours, the patient was sedated using propofol and remifentanil. Hereafter, sedation was reduced at the earliest convenience to evaluate the level of consciousness. The temperature of the patients who remained comatose was actively kept at ≤37.5 °C until 72 hours after the cardiac arrest. Neurological prognosis was evaluated at a minimum of 72 h after cardiac arrest in accordance with the 2021 European guidelines.^5^ As part of the prognostic examinations, the PLR was assessed by pupillometry in conjunction with SSEP when it was performed >48 h after cardiac arrest.

### Procedures

The PLR was examined bilaterally, using a handheld device for automated infrared pupillometry (NeurOptics NPi®-200 Pupillometer; Neuroptics Ltd, Irvine, CA, USA). The pupillometer emits a light impulse with fixed intensity and duration, during which the infrared camera captures multiple parameters. To ensure uniform distance from pupil to pupillometer a single-use plastic chin guard (SmartGuard®; Neuroptics Ltd, Irvine, CA, USA) is used attached to the pupillometer. The measures provided by the pupillometer are maximum and minimum pupil diameter (millimetres), proportional change in pupil size (percentage), constriction velocity, average and maximum constriction velocity (millimetres/second), latency of constriction (seconds), dilation velocity (millimetres/second) and the NPi.^19^ The NPi is an index of the pupil reactivity to light stimulation calculated by a built-in algorithm based on the other parameters measured by the pupillometer. The index is scaled from 0 to 5 where a higher score corresponds to a higher reactivity. A score < 3 is considered to signify an abnormally slow response and 0 signifies the absence of pupillary reactivity.^14^ A score ≤ 2 is associated with a poor neurologic outcome in comatose survivors of cardiac arrest.^9^, ^13^, ^20^ NPi values can vary between a patient's eyes. In accordance with Riker et al., who argued that a unilateral change reflects a clinical state, we used the lower value in our analysis of such cases.^12^

Somatosensory evoked potentials were recorded on a standard electrodiagnostic system (KeyPoint G4, software v 2.32; Natus Technology Europe Gmbh). The median nerve was stimulated with a 2.7 Hz frequency impulse with a 0.2 ms stimulus duration in two sets of at least 500 repetitions using bipolar surface electrodes on the forearm. The filter bandpass was 10 Hz to 2 kHz. Detecting surface electrodes (Ag/AgCl) were placed on Erb's point, the cervical spine (C7) and 2 cm posterior to C3 and C4 (C3′ and C4′, respectively) on the contralateral side. The definition of an N20 signal classified as a present response was a N20-P25 amplitude equal to or larger than 0.30 µV. The maximum accepted level of baseline noise level was 0.25 µV and if it was higher, a bolus of rocuronium muscle relaxant was administered after approval by the physician responsible for the patient.^21^, ^22^ The SSEP recording was interpreted by a specialist or resident in clinical neurophysiology and the N20 signal was classified as either bilaterally absent or present.^15^

### Clinical variables

Demographic and clinical data including the full assessment of the neurological prognosis with NPi and SSEP were retrieved from medical records including the ICU monitoring system. Possible confounding variables were defined as; diabetes, pre-existing neurologic conditions and pre-existing ophthalmological conditions, administration of remifentanil and/or propofol during examination with the pupillometry. Pre-existing neurologic conditions were defined as previous stroke, transient ischemic attack (TIA) and/or polyneuropathy. The neurological outcome was defined according to the Modified Rankin scale (MRs) at hospital discharge as assessed by occupational therapist notes in the medical record. Survival outcome was defined as 30-day survival.

### Statistical analysis

The sample size was calculated from NPi values with median and interquartile range (IQR) categorised according to neurological outcome by Oddo et al. and an estimated prevalence of absent cortical SSEP response by Moseby-Knappe et al.^6^, ^9^ To find a significant difference in NPi of 0.7 between patients with preserved and bilaterally absent SSEP N20 response with a power of 95%, 45 patients were needed, assuming a 2:1 allocation and unequal SD in the groups (0.37 and 0.67 respectively, calculated from the IQR in reference 9) and significance level 0.01. To account for uncertainty within these estimates, we aimed to include 50 patients. Continuous variables are expressed as median (interquartile range) and categorical variables are expressed as proportions (percentage). The patients without N20 SSEP response were compared to the patients with N20 SSEP response using group statistics. The NPi threshold value resulting in an FPR < 5% for bilaterally absent SSEP N20 response was identified from a tabulation of several threshold values. A receiver operating characteristics (ROC) curve was used to evaluate the capacity of NPi to predict bilaterally absent SSEP N20 response.

## Results

**Fig. 1:**
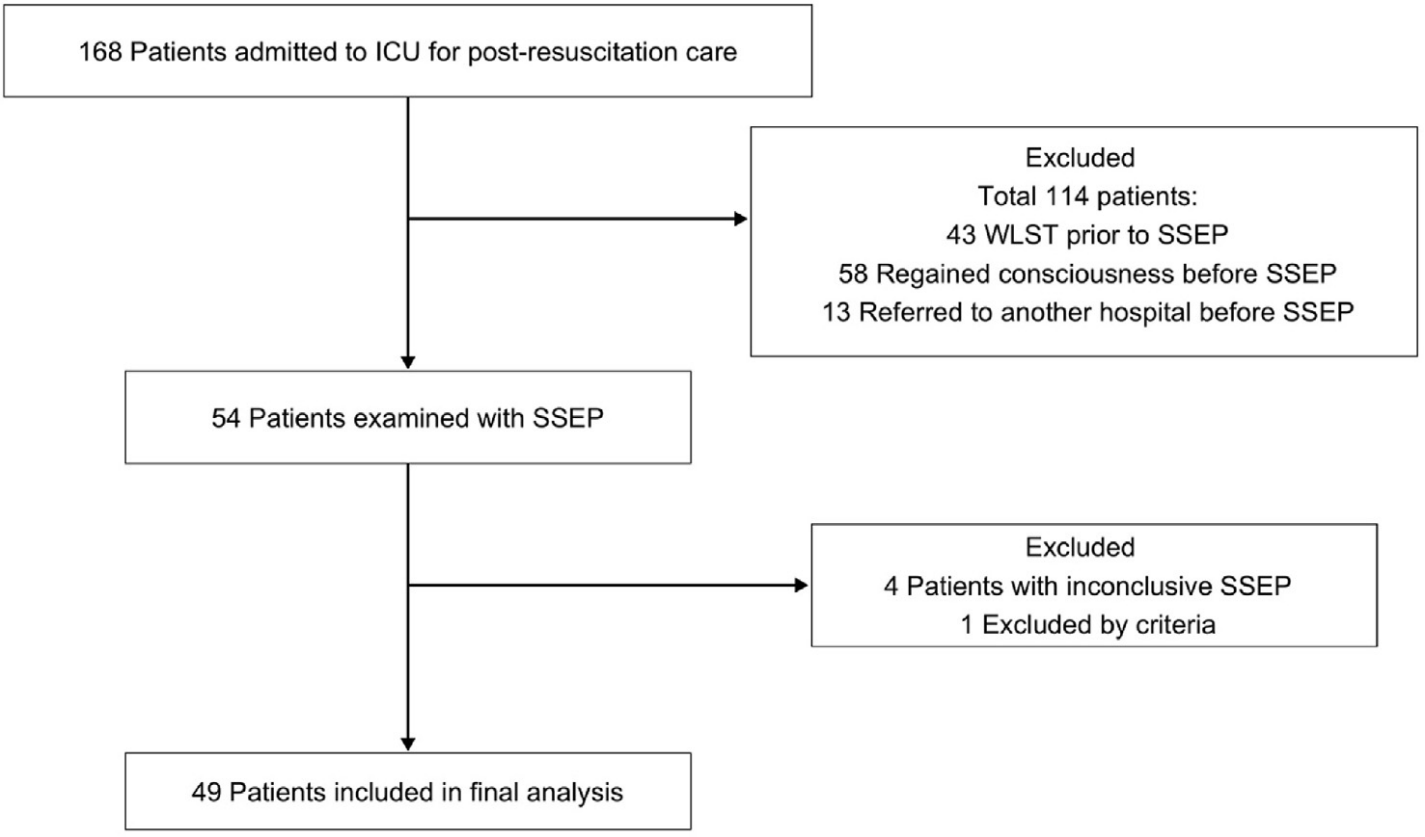
Flowchart of the study inclusion.

During the study period a total of 168 eligible patients were admitted to the general ICU. Of these, 54 patients were examined with SSEP. Five patients were excluded after SSEP; four had inconclusive examinations due to too high baseline noise level, or weak/absent peripheral signals despite repeated attempts to improve the quality of the registration and one patient was later found to have sustained a traumatic brain injury in addition to the HIE. The final analysis included 49 patients (Fig. 1).

None of the patients had any known ophthalmological disorder. The overall mean (SD) age for the entire cohort was 60.3 (16.9) years, and 71% were female. The patients with absent SSEP N20 response had a higher proportion of diabetes mellitus and had a 0% 30-day survival rate as opposed to 26% in the group with preserved SSEP N20 response (Table 1).

**Table 1:** Baseline characteristics.

	Overall	N20 bilaterally absent	N20 present	Missing data, %
*n*	49	22	27	
Age, mean (SD)	60.3 (16.9)	59.9 (18.4)	60.6 (16.0)	0.0
Sex, Female, *n* (%)	35 (71)	14 (64)	21 (78)	0.0
BMI, mean (SD)	28.9 (6.5)	30.2 (7.8)	27.9 (5.4)	6.1
Comorbidities, *n* (%)				
Pre-existing neurologic condition	6 (12)	3 (14)	3 (11)	0.0
Diabetes mellitus	12 (24)	9 (41)	3 (11)	0.0
Cardiac arrest characteristics, *n* (%)				
In-hospital cardiac arrest	9 (18)	1 (5)	8 (30)	0.0
Bystander CPR	40 (82)	16 (73)	24 (89)	4.1
Shockable first rhythm	17 (35)	5 (23)	12 (44)	4.1
Presumed cardiac cause of cardiac arrest	23 (47)	8 (36)	15 (56)	0.0
Clinical neurological examination after 72 hours				
Manual Pupillary Light Reflex, *n* (%)				2.0
Bilaterally absent	9 (19)	7 (32)	2 (8)	
Corneal reflex, *n* (%)				6.1
Bilaterally absent	13 (28)	9 (45)	4 (15)	
Biomarkers, mean (SD)				
NSE 24 h	67.9 (62.8)	103.3 (81.3)	41.0 (19.0)	10.2
NSE 48 h	133.0 (123.3)	216.5 (134.1)	70.4 (65.2)	14.3
NSE 72 h	156.3 (168.6)	256.9 (147.1)	95.1 (153.0)	24.5
Electroencephalogram (EEG), *n* (%)				0.0
Highly malignant pattern	15 (31)	12 (55)	3 (11)	
Brain imaging, *n* (%)				0.0
Anoxic/ischemic brain injury	25 (51)	12 (55)	13 (48)	
Neurologic/survival outcome				
Modified Rankin Scale (mRS) at hospital discharge, *n* (%)				6.1
mRS 0–3	6 (12)	0 (0)	6 (22)	
mRS 4–6	41 (84)	22 (100)	19 (70)	
30-day survival, *n* (%)	7 (14)	0 (0)	7 (26)	2.0

Data are presented as count (percentage) and mean (standard deviation). Pre-existing neurologic condition were defined as previous stroke, transient ischemic attack (TIA) and/or polyneuropathy.

The number of patients with ongoing Propofol infusion during SSEP was larger in the group with preserved SSEP N20 response. The number of patients with remifentanil infusion during SSEP and the mean doses for this infusion were similar in both groups (Table 2).

**Table 2:** Analgosedation during Somatosensory evoked potentials examination.

	N20 bilaterally absent	N20 present
SSEP performed, n (%)	22 (45)	27 (55)
Propofol infusion at time of SEP, *n* (%)	8 (36)	14 (52)
Propofol dose at time of SEP, mg/kg/h, mean (SD)	1.8 (1.2)	1.8 (0.9)
No Propofol infusion at time of SSEP, *n* (%)	14 (64)	13 (48)
Time from last dose of Propofol to SSEP, minutes, mean (SD)	326.0 (437.8)	196.9 (418.4)
Remifentanil infusion at time of SSEP, *n* (%)	13 (59)	14 (52)
Remifentanil dose at time of SEP, µg/kg/min, mean (SD)	0.2 (0.3)	0.3 (0.6)
No Remifentanil infusion at time of SSEP, *n* (%)	9 (41)	13 (48)
Time from last dose of Remifentanil to SSEP, minutes, mean (SD)	332.0 (528.6)	117.3 (119.6)

A bolus of rocuronium muscle relaxant was given to 14 (64%) patients in the group with absent SSEP N20 response and to 8 (32%) patients with preserved SSEP N20 response.

**Fig. 2:**
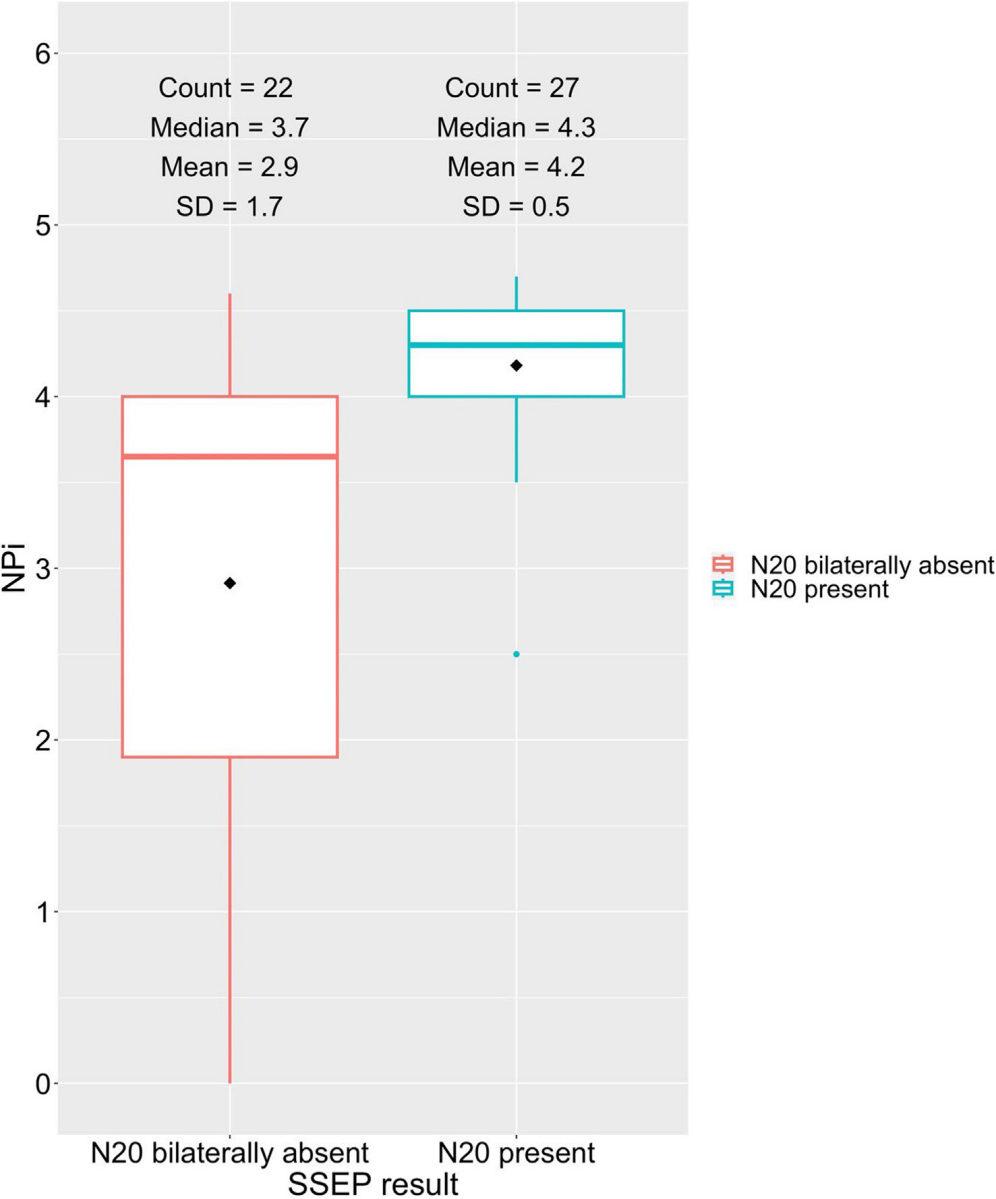
The difference in Neurological pupil index (NPi) values between patients with N20 signal present and N20 signal bilaterally absent The group mean for NPi is marked with a black diamond in the boxplot. The mean difference is 1.3 (95% CI 0.5–2.1).

Forty-one patients had different NPi values between their eyes. The mean (SD) NPi in the group with absent SSEP N20 response was 2.9 (1.7) and 4.2 (0.5) in the group with preserved SSEP N20 response (Fig. 2). The mean difference between the two groups was 1.3 (95% CI 0.5 – 2.1). Among the patients with preserved SSEP N20 response, 1 (3.7%) had an NPi score ≤ 3 and 0 (0.0%) a score ≤2. Among the patients with absent SSEP N20 response, 6 (27.3%) had an NPi score ≤ 3 and 6 (27.3%) a score ≤ 2.

**Fig. 3:**
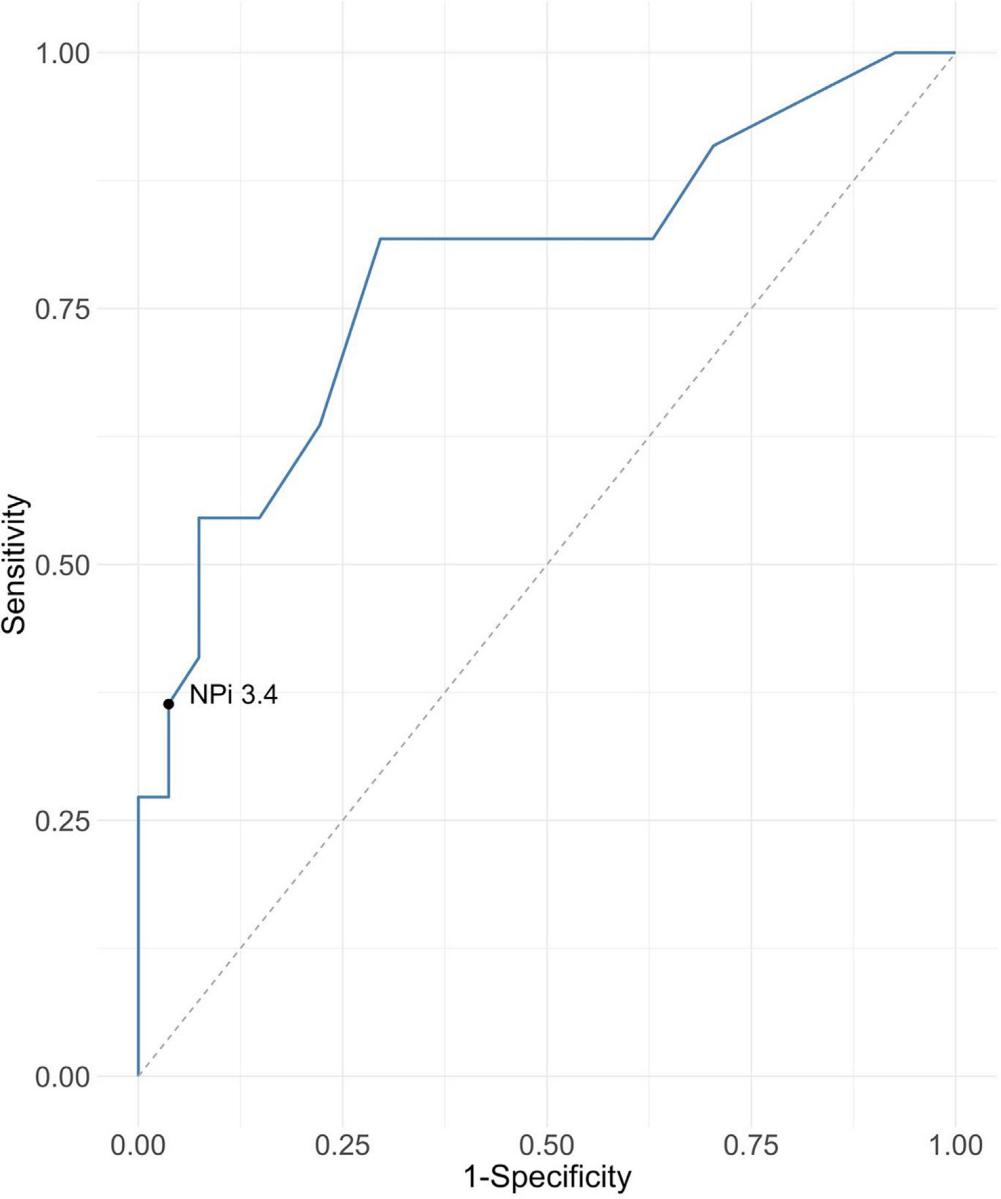
The capacity of Neurological pupil index (NPi) to predict the bilateral absence of Somatosensory evoked potentials (SSEP) response, presented with a receiver operating characteristic (ROC) curve with AUC = 0.79 (95% CI 0.65–0.92). Among the values yielding a false positive rate <5%, NPi 3.4 was associated with the highest sensitivity, 36%.

Among the values yielding a false positive rate < 5%, NPi 3.4 was associated with the highest sensitivity, 36% (95% CI 18–55) (Table 3). A receiver operating curve for the capacity of NPi to predict an absent N20 response had an area under the curve (AUC) of 0.79 (95% CI 0.65–0.92) (Fig. 3).

**Table 3:** The predictive capacity of different Neurological pupil index thresholds for bilateral absence of Somatosensory evoked potentials response.

NPi	PPV, % (95% CI)	NPV, % (95% CI)	Specificity, % (95% CI)	Sensitivity, % (95% CI)	TP, *n*	FP, *n*	TN, *n*	FN, *n*
4.6	47 (45–50)	100 (100–100)	7 (0–19)	100 (100–100)	22	25	2	0
4.5	49 (44–54)	86 (50–100)	19 (4–33)	95 (86–100)	21	22	5	1
4.4	51 (45–58)	80 (56–100)	30 (15–48)	91 (77–100)	20	19	8	2
4.3	51 (43–62)	72 (50–93)	37 (19–56)	82 (64–95)	18	17	10	4
4.2	58 (48–70)	78 (61–94)	52 (33–70)	82 (64–95)	18	13	14	4
4.1	64 (53–79)	81 (67–95)	63 (44–81)	82 (64–95)	18	10	17	4
4.0^a^	69 (57–83)	83 (69–95)	70 (52–85)	82 (64–95)	18	8	19	4
3.9	70 (53–88)	72 (61–85)	78 (59–93)	64 (41–82)	14	6	21	8
3.8	75 (57–93)	70 (60–81)	85 (70–96)	55 (32–73)	12	4	23	10
3.7	87 (67–100)	71 (63–82)	93 (81–100)	55 (32–77)	12	2	25	10
3.6	86 (64–100)	69 (62–80)	93 (81–100)	50 (32–73)	11	2	25	11
3.5	83 (60–100)	66 (59–75)	93 (81–100)	41 (23–64)	9	2	25	13
3.4^b^	90 (64–100)	65 (58–73)	96 (89–100)	36 (18–55)	8	1	26	14
3.2	89 (63–100)	63 (57–71)	96 (89–100)	32 (14–50)	7	1	26	15
2.8	86 (50–100)	62 (57–69)	96 (89–100)	27 (9–45)	6	1	26	16
2.0^c^	100 (100–100)	63 (57–69)	100 (100–100)	27 (9–45)	6	0	27	16
0.7	100 (100–100)	61 (56–68)	100 (100–100)	23 (5–41)	5	0	27	17

PPV = positive predictive value. NPV = negative predictive value. TP = True positive. FP = False positive. TN = True negative. FN = False negative.

a Optimal threshold based on Youden index.

b NPi threshold with FPR < 5% and highest sensitivity 36%.

c Npi ≤ 2 was associated with PPV 100%.

## Discussion

Our reasoning was that a certain level of depressed PLR response corresponding to a measured NPi value would be predictive of bilaterally absent cortical SSEP N20 response in patients remaining comatose > 48 hours after resuscitation from cardiac arrest. In a cohort of 49 patients, we demonstrated that NPi < 3.4 was predictive of a bilateral absence of the SSEP N20 response with a sensitivity of 36% and FPR below 5%. In comparison with previous studies that investigated the optimal NPi threshold value indicating poor prognosis or increased intracranial pressure, 3.4 is a higher threshold value. Chen et al. stated that NPi < 3 represents an abnormally slow PLR and corresponded to an increased intracranial pressure in patients treated in neurologic intensive care units.^14^ In a multicentre study by Oddo et al, NPi ≤ 2 was found predictive of poor neurologic outcome 3 months after cardiac arrest.^9^ Subsequent studies also indicated that a threshold value for NPi to predict poor neurologic outcome was approximately NPi ≤ 2.^13^, ^20^ However, in these reports, the authors presented a threshold value with 100% positive predictive value (PPV). We chose to define a threshold value with a reasonable FPR of <5%, noting that the 95% CI would include a maximum FPR of 11% for predicting bilateral absence of the SSEP N20 response. In line with the previously mentioned studies, an NPi < 2 in our patients showed a PPV of 100% for a bilaterally absent SSEP N20 response, but the sensitivity was limited to 27%.

In the study by Oddo et al, NPi ≤ 2 predicted poor outcome in a number of patients with a preserved SSEP N20 response. Also, the combination of NPi ≤ 2 and absent SSEP N20 response improved the sensitivity for predicting poor neurologic outcome.^9^ However, in a post-hoc analysis, the authors found that 27% of patients with bilaterally absent SSEP N20 response, also had NPi ≥2.^11^ Another study investigating the concordance between the different modalities for prognostication found that 46% of patients with bilaterally absent SSEP N20 response also reached an NPi below 2 within the first 48 hours.^7^ In these studies, the overall mortality rate and the proportion of patients with unfavourable outcome was lower than in our study which could be explained by our selection of patients still comatose later than 48 hrs after cardiac arrest. Yet, these varying results suggest that hypoxic/ischemic injury does not necessarily follow a sequence defined by the neuroanatomical structures as we reasoned, but that the anatomical location of the lethal lesions may vary. In a post-hoc analysis of the multicentre study by Oddo et al., the AUC for NPi capacity to predict bilaterally absent SSEP N20 response was 0.68 (95% CI 0.61–0.76).^11^ This is lower than the AUC from our ROC-analysis, but it showed a wider 95% confidence interval as a result of our smaller sample size.

Our choice to define an absent SSEP N20 response by a signal amplitude below 0.30 µV was based on a nationally agreed threshold value intended to maximize the specificity of the examination. Earlier studies have found varying lower limits of N20-P25 amplitude (0.45–1.00 µV) in patients with good neurologic outcome.^21^, ^23^, ^24^, ^25^, ^26^ In a recent study, a 0.50 µV threshold was associated with a sensitivity of 42.9% and still a high specificity for poor outcome with only one patient regaining consciousness and achieving a good outcome.^27^ Therefore, it is not excluded that the application of a higher threshold than the 0.30 µV that we used could increase the sensitivity for poor outcome in our material, but we preferred to keep a margin to the lowest N20-P25 amplitude 0.45 µV compatible with good outcome as described in the literature.^26^

### Strengths and limitations

Strengths of this study included the data sampling from everyday practice, which enhances its internal validity. Pupillometry was performed by two of the authors with special training in using the device, and with no role in the clinical assessment of the neurological prognosis. The SSEP was performed by a technician in clinical neurophysiology trained in SSEP and the registration was analysed by a trained specialist or resident in clinical neurophysiology.

Limitations include the lack of a validation cohort, and the results should be regarded as hypothesis-generating. In NPi-testing there may be confounding by diabetes retinopathy and diabetes autonomic neuropathy that have been shown to affect the PLR.^28^, ^29^ In our study the group with absent SSEP N20 response consisted of a larger number of patients with diabetes compared to the group with preserved SSEP N20 response. Nevertheless, data regarding the presence of diabetes retinopathy or diabetes autonomic neuropathy were not available. However, comparison to important NPi studies would have been precluded anyway as they did not present these data.^9^, ^10^, ^12^, ^13^, ^20^ Additional variables to be considered as potential confounding variables were the sedative drugs used in the ICU, but we showed a distribution counterintuitive to one that could affect the results. In addition, evidence suggests that propofol and remifentanil do not affect SSEP response and PLR to a clinically significant degree.^30^, ^31^ Finally, the relatively small study population and single centre investigation was a limitation of this study.

## Conclusion

In this cohort with patients who remained comatose >48 hours after cardiac arrest, Neurological Pupil index values <3.4 predicted bilateral loss of SSEP N20 response with a false positive rate of <5%. However, this value differs from previous investigations assessing the concordance of NPi and bilateral absence of N20 response on SSEP and it needs to be validated in a larger cohort. If confirmed, an NPi threshold may be clinically applied in settings where SSEP is unavailable.

## CRediT authorship contribution statement

**Meena Thuccani:** Writing – review & editing, Writing – original draft, Visualization, Software, Project administration, Methodology, Investigation, Formal analysis, Data curation, Conceptualization. **Sara Joelsson:** Writing – review & editing, Visualization, Methodology, Investigation, Formal analysis, Data curation, Conceptualization. **Linus Lilja:** Writing – review & editing, Visualization, Project administration, Methodology, Investigation, Formal analysis, Conceptualization. **Axel Strålin:** Writing – review & editing, Visualization, Project administration, Investigation, Formal analysis. **Josefin Nilsson:** Writing – review & editing, Visualization, Investigation, Formal analysis. **Petra Redfors:** Writing – review & editing, Visualization, Formal analysis. **Araz Rawshani:** Writing – review & editing, Visualization, Formal analysis. **Johan Herlitz:** Writing – review & editing, Visualization, Formal analysis. **Peter Lundgren:** Writing – review & editing, Visualization, Resources, Funding acquisition, Formal analysis. **Christian Rylander:** Writing – review & editing, Writing – original draft, Visualization, Supervision, Resources, Project administration, Methodology, Investigation, Formal analysis, Conceptualization.

## Declaration of competing interest

The authors declare that they have no known competing financial interests or personal relationships that could have appeared to influence the work reported in this paper.
